# Strengthening of Ceramic-based Artificial Nacre via Synergistic Interactions of 1D Vanadium Pentoxide and 2D Graphene Oxide Building Blocks

**DOI:** 10.1038/srep40999

**Published:** 2017-01-19

**Authors:** Andrea Knöller, Christian P. Lampa, Felix von Cube, Tingying Helen Zeng, David C. Bell, Mildred S. Dresselhaus, Zaklina Burghard, Joachim Bill

**Affiliations:** 1 Institute for Materials Science, University of Stuttgart, Heisenbergstr.3, 70569 Stuttgart, Germany; 2 John A. Paulson School of Engineering and Applied Sciences, Harvard University, Cambridge, MA 02138, USA; 3 Department of Electrical Engineering and Computer Sciences, Massachusetts Institute of Technology, Cambridge, MA 02139, USA; 4 Department of Chemistry and Chemical Engineering, School of Chemical Engineering and Environment, Beijing University of Technology, Beijing, 100124, P.R. China; 5 Department of Physics, Massachusetts Institute of Technology, Cambridge, MA 02139, USA

## Abstract

Nature has evolved hierarchical structures of hybrid materials with excellent mechanical properties. Inspired by nacre’s architecture, a ternary nanostructured composite has been developed, wherein stacked lamellas of 1D vanadium pentoxide nanofibres, intercalated with water molecules, are complemented by 2D graphene oxide (GO) nanosheets. The components self-assemble at low temperature into hierarchically arranged, highly flexible ceramic-based papers. The papers’ mechanical properties are found to be strongly influenced by the amount of the integrated GO phase. Nanoindentation tests reveal an out-of-plane decrease in Young’s modulus with increasing GO content. Furthermore, nanotensile tests reveal that the ceramic-based papers with 0.5 wt% GO show superior in-plane mechanical performance, compared to papers with higher GO contents as well as to pristine V_2_O_5_ and GO papers. Remarkably, the performance is preserved even after stretching the composite material for 100 nanotensile test cycles. The good mechanical stability and unique combination of stiffness and flexibility enable this material to memorize its micro- and macroscopic shape after repeated mechanical deformations. These findings provide useful guidelines for the development of bioinspired, multifunctional systems whose hierarchical structure imparts tailored mechanical properties and cycling stability, which is essential for applications such as actuators or flexible electrodes for advanced energy storage.

Investigation of biomaterials, such as nacre, helps scientists to understand the correlation between their structure and mechanical properties^1^. These materials are characterized by a well-defined composition and a high degree of hierarchical organization, including (i) the combination of materials that strongly differ in Young’s modulus, (ii) nanostructures with a high structural aspect ratio, (iii) optimized content ratio and (iv) their assembly into an alternating layer architecture. These characteristics together account for nacre’s extraordinary combination of mechanical strength, stiffness and toughness^2^,^3^,^4^,^5^,^6^,^7^.

In recent years, various attempts were made to fabricate predominantly binary systems in the form of thin-films^5^,^8^,^9^,^10^,^11^,^12^,^13^,^14^,^15^ or even fibres^16^,^17^,^18^,^19^, which exhibit hierarchical, layered structures. The goal is to achieve functional materials with mechanical properties comparable or even superior to those of nacre. However, a closer look at nacre reveals that it does not just consist of two components, namely 2D aragonite platelets embedded into a biopolymer matrix. In fact, there exists a third phase in the form of a 1D nanofibrillar chitin network^20^. The interaction between the 1D and 2D building blocks can lead to sliding effects, crack deflection and crack bridging, thereby enhancing the strength and toughness of such composite materials in a synergistic manner^21^,^22^. Recently, several studies have addressed the design and mechanical characterization of ternary artificial nacre^23^,^24^,^25^,^26^,^27^,^28^. For instance, Wang *et al*.^24^ reported a hierarchical, layered composite material composed of 2D clay and 1D nanofibrillar cellulose embedded into a poly(vinyl alcohol) (PVA) matrix. This ternary system mimics nacre’s structure more closely, and hence features superior mechanical properties in comparison to the corresponding binary systems.

In general, such polymer- or graphene-based ternary systems stand out due to excellent tensile strength and/or toughness. However, owing their comparably soft matrix material, they exhibit only a low Young’s modulus^25^,^26^,^27^,^28^. In order to achieve a ternary system that combines good tensile strength with high stiffness and form stability, designing a ceramic-based ternary system represents a promising approach. Our previous work addressed the bioinspired fabrication and characterization of V_2_O_5_ nanofibre papers^29^. This binary system, which is composed of hierarchically arranged 1D V_2_O_5_ nanofibres and intercalated water molecules, shows excellent mechanical properties due to the presence of a hydrogen-bonded network. The high aspect ratio and mechanical flexibility of the V_2_O_5_ nanofibres render such paper into a close-to-ideal ceramic-based matrix. In order to realize a ternary system with differently shaped building blocks, in the present work 2D GO nanosheets are incorporated into the layered V_2_O_5_ nanofibre matrix. In the resulting composites, smooth structural integration is expected due to GO’s 2D structure and the presence of oxygen-containing functional groups located on the basal planes. In contrast to previous reports^24^,^25^,^27^, such a ternary system features an “inverse” composition with a small amount of soft component embedded into a comparatively harder matrix. The interaction between the different building blocks should lead to a unique combination of high tensile strength, good toughness, as well as excellent stiffness in a synergistic fashion. We fabricated the layered composite papers *via* a bioinspired self-assembly approach and used nanoindentation and nanotensile tests to mechanically characterized them as a function of the GO content. In addition, cyclic nanotensile tests testify the remarkable mechanical cycling stability of the composite material.

## Results and Discussion

### Hierarchical Structuring

**Figure 1:**
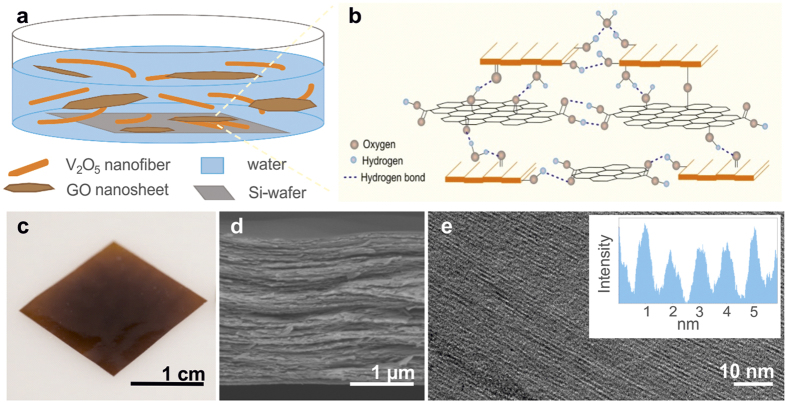
Self-assembling of V_2_O_5_ and GO building blocks into hierarchical microstructures. Schematic depiction of (**a**) sample fabrication *via* a self-assembly approach, and (**b**) the self-assembly of the components, which is promoted by hydrogen bond formation. (**c**) Optical image of a paper sample (containing 0.5 wt% GO). (**d**) SEM micrograph showing the layered microstructure of the 2.5 μm thick paper. (**e**) HRTEM cross-sectional image revealing a parallel lamella alignment with an interlayer distance of approximately 1 nm, as deduced from the intensity profile (insert).

Hydrated V_2_O_5_ nanofibres were obtained through polycondensation of vanadate ions in aqueous solution under acidic conditions^30^. Atomic force microscopy (AFM) of thus obtained nanofibres reveals a high aspect ratio with an average length in micrometre range. From AFM height profiles, the fibres thickness is estimated to be 1.5 nm (Supplementary Fig. S1). This value is in good agreement to that for single nanofibres composed of two corrugated single sheets made of square VO_5_ pyramidal units, having oxygen-functionalities on their surface and water molecules located in between^30^,^31^. GO nanosheets were produced using a modified Hummers’ method, in which graphite is chemically oxidized by KMnO_4_^32^. The resulting GO nanosheets have an average thickness of 1.5 nm (Supplementary Fig. S1), in agreement with the value reported for single-layered GO sheets^33^. This thickness can be attributed to oxygen-containing functional groups linked to sp^3^-hybridized carbon atoms, which lead to a distorted honeycomb structure. The average lateral size of the GO nanosheets is several tens of micrometres. In general, improving the mechanical properties of composite materials requires both, a high aspect ratio of the components, and their highly ordered layered arrangement^5^. Both these requirements are fulfilled for the present V_2_O_5_ nanofibres and GO nanosheets, which in addition display similar thickness. In order to investigate the influence of the incorporated GO on the mechanical properties of V_2_O_5_ nanofibre papers, we fabricated composite materials containing 0.5, 2.5 and 5.0 wt% GO (for more details see the supporting information)^29^. The combined self-assembly of V_2_O_5_ nanofibres and GO nanosheets from aqueous solution (Fig. 1a) into a regular film architecture is likely to be facilitated by the formation of the hydrogen bonds between water molecules, the hydroxyl- and oxo-groups of the V_2_O_5_^30^ and oxygen-containing functional groups of the GO^34^,^35^ (Fig. 1b), similar to the coassembly of GO nanosheets and Na_0.44_MnO_2_ nanowires^36^. Lateral hydrogen bonds can be formed between the hydroxyl- and carboxyl groups at the edges of the V_2_O_5_ nanofibres and GO nanosheets, respectively. In the vertical direction, also the hydroxyl- and oxo-groups on the V_2_O_5_ nanofibre surface can participate in hydrogen bonding and oxygen bridging. Since the basal planes comprise a higher density of functional groups, a vertical stacking of V_2_O_5_ nanofibres and GO nanosheets is favourable. The resulting 2.5 μm thick composite papers (Fig. 1c) feature a transparent, red-brown colour and excellent flexibility. Inspection of the composite’s structure by scanning electron microscopy (SEM) demonstrated that the self-assembly results in a well-ordered layer structure (Fig. 1d), similar to that of papers obtained by vacuum filtration^37^,^38^. Moreover, a high degree of vertical ordering can be seen down to the nanometre scale. Specifically, high resolution transmission electron microscopy (HRTEM) of a cross-section lamella evidences a uniform stacking (Fig. 1e) with an interlayer distance of about 1 nm (insert of Fig. 1e). The observed interlayer distance is verified by using X-ray diffraction (XRD) peak position analysis. (Supplementary Fig. S2). All three composites showed similar peak positions with a calculated interlayer distance of ~1.04 nm, which is correlated to the V_2_O_5_ hydration state^30^. It thus follows that in all the samples, the V_2_O_5_ nanofibres have similar water content (about 1.4 H_2_O molecules per V_2_O_5_) and binding properties. The absence of GO nanosheet agglomerates indicates that the GO is vertically well distributed within the V_2_O_5_ nanofibre matrix. This conclusion is supported by EDS data gained via cross-sectional TEM of the lamella (Supplementary Fig. S3).

**Figure 2:**
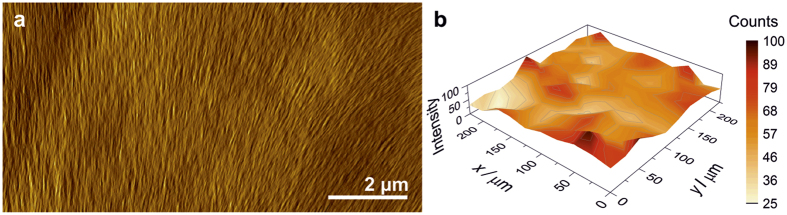
V_2_O_5_ nanofibre alignment and vertical GO distribution. (**a**) AFM image of the sample containing 0.5 wt% GO. The alignment of the V_2_O_5_ nanofibres is slightly disturbed by the GO nanosheets located beneath. (**b**) Raman map displaying the absolute intensity of the G-band of GO less the measured background, revealing its homogeneous lateral distribution over several 100 μm^2^.

Contrary to vacuum filtrated papers, self-assembly not only ensures good vertical layering, but also pronounced lateral alignment of the nanofibres, as has been documented for self-assembled, V_2_O_5_ nanofibre papers^29^. The AFM image in Fig. 2a displays domains with aligned V_2_O_5_ nanofibres and small, local corrugations presumably caused by GO nanosheets below the sample’s surface. Similar observations were made by SEM over larger scan areas (Supplementary Fig. S4). The presence and distribution of GO sheets below the surface could be confirmed by Raman spectroscopy (Fig. 2b and Supplementary Fig. S5), by exploiting that the Raman peaks of V_2_O_5_^39^ and GO^40^ occur at different energies (in the region up to 1000 cm^−1^ for V_2_O_5_ and the D and G bands of GO at 1348 cm^−1^ and 1592 cm^−1^, respectively). Scans of several 100 μm^2^ revealed a laterally homogeneous distribution of the GO nanosheets. The spatial modulation of intensity most likely originates from a variation in the vertical distribution of the nanosheets.

### Mechanical Performance

**Figure 3:**
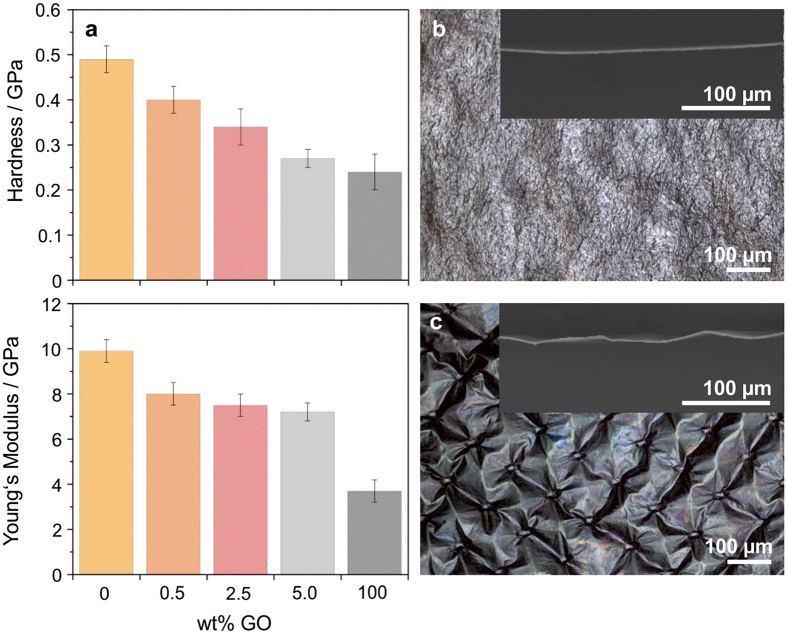
Out-of-plane mechanical characterization. (**a**) Hardness and Young’s modulus of papers made exclusively of V_2_O_5_ nanofibres, or GO nanosheets, as well as the corresponding composite papers (0.5, 2.5 and 5.0 wt% GO), as determined by nanoindentation. The error bars in **a** describe the standard deviation of the displayed values. Optical and cross-sectional SEM images (insets) of nanofibre papers with (**b**) 0.5 wt% GO and (**c**) 5.0 wt% GO.

In order to investigate the out-of-plane mechanical performance of the composites in dependence of the GO content, nanoindentation tests were performed and hardness and Young’s modulus were determined (Fig. 3a). The pristine V_2_O_5_ nanofibre paper exhibited an average hardness of 0.49 GPa. Upon addition of GO, the hardness gradually decreased with increasing amount of incorporated GO (which showed a hardness of 0.24 GPa). Thus, the paper experiences out-of-plane softening as a consequence of physical mixing of hard and soft components. An opposite trend was reported by Das *et al*.^41^ who investigated the mechanical properties of PVA enriched with few-layer graphene by nanoindentation. In their study, the initially low elastic modulus and hardness of the soft PVA increased due to the incorporation of the comparatively harder graphene. For the present samples, softening was detected by light microscopy and low magnification SEM imaging of cross-sections. Papers with a GO content of 0.5 wt%, which appeared to be harder and stiffer, exhibited a smooth and uniform structure on both the microscopic and macroscopic scale (Fig. 3b and Supplementary Fig. S6). In contrast, papers containing 5.0 wt% GO were more flexible and easily adapted the mesh-like structure of the underlying sieve (used for drying the papers) and therefore appeared strongly wrinkled (Fig. 3c). Like the hardness, also the Young’s modulus gradually decreased with increasing GO content from the initially 9.9 GPa for V_2_O_5_ nanofibre papers to 3.7 GPa for GO papers (Fig. 3a). A similar trend was reported by Tritschler *et al*.^42^, who combined V_2_O_5_ ribbons with a much softer liquid crystal polymer as “glue”.

**Figure 4:**
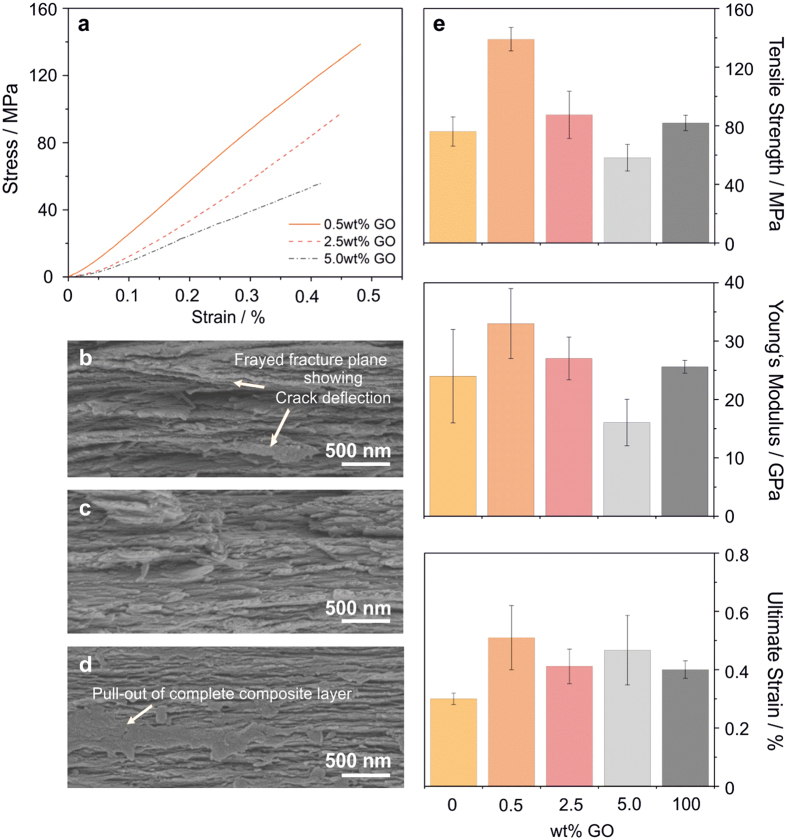
In-plane mechanical characterization. (**a**) Stress-strain curves of the investigated composite papers, acquired by nanotensile tests. SEM images of the fracture surface of papers with (**b**) 0.5 wt%, (**c**) 2.5 wt% and (**d**) 5.0 wt% GO. (**e**) Comparison of the mechanical properties of V_2_O_5_ nanofibre papers^29^, GO papers^11^ and the investigated composite papers. The error bars in (**e**) describe the standard deviation of the displayed values.

In addition, nanotensile tests were performed to evaluate the papers’ in-plane mechanical performance. Figure 4a presents representative stress-strain curves for the three investigated GO contents. A pronounced modulation of mechanical performance as a function GO content is apparent from Supplementary Table S1. The papers with 0.5 wt% GO exhibit the best mechanical properties. Increasing the GO content to 2.5 and 5.0 wt% resulted to a continuous decrease in tensile strength, Young’s modulus and toughness. The corresponding SEM cross-sectional images reveal stiff, frayed fracture planes in the case of composite papers with 0.5 wt% (Fig. 4b and Supplementary Fig. S4) and 2.5 wt% GO (Fig. 4c and Supplementary Fig. S4), whereas papers with 5.0 wt% GO show slipping and pullouts of whole, flexible composite layers (Fig. 4d and Supplementary Fig. S4). The mechanical properties of the different samples are directly contrasted in Fig. 4e. Comparison with the values of V_2_O_5_ nanofibre-29 and GO papers^11^ underscores the excellent mechanical performance of the composite containing 0.5 wt% GO. The V_2_O_5_ nanofibre matrix exhibits a tensile strength of about 76 MPa. Upon incorporation of 0.5 wt% GO, which itself reaches a value of 82 MPa in the form of papers, the tensile strength almost doubles (139 MPa). The Young’s modulus (33 GPa), ultimate strain (0.5 %) and toughness (325 kJ/m^3^) increase by a factor of 1.4, 1.7 and 4.3, respectively, as compared to the bare V_2_O_5_ nanofibre matrix (24 GPa, 0.3 % and 76 kJ/m^3^). This enhancement of mechanical performance is owed to the significant interaction between the components. As illustrated in Fig. 1b, the V_2_O_5_ nanofibres and GO nanosheets can be linked *via* hydrogen bonds and oxygen bridges, which provide resilience against in-plane mechanical deformation. When mechanical stress is applied, the hydrogen bonds between the components break and reform slightly displaced, analogue to the findings of Sinko and Keten^43^. This so-called “stick-slip effect” leads to enhanced flexibility and fracture resistance. Another factor contributing to the improved fracture resistance is the larger surface area of the GO nanosheets in comparison to the V_2_O_5_ nanofibres. According to theoretical and experimental studies, incorporation of 2D sheets into a polymer matrix is more efficient in crack deflection than the incorporation of 1D fibres, due to a larger interface between the matrix and the incorporated phase^44^. For the present V_2_O_5_ nanofibre-based ternary system, the crack deflection is characterized by the fracture plane’s frayed surface. Moreover, the V_2_O_5_ nanofibres can interlock with the slightly wrinkled, distorted honeycomb structure of the GO nanosheets and thereby effectively bridge cracks. This bridging is accompanied by pull-outs of nanofibre bundles during fracture of the composite material.

The combination of stick-slipping, crack deflection and crack bridging enables the best mechanical enhancement observed for composite papers with 0.5 wt% GO. Wang *et al*.^24^ successfully demonstrated that combining 2D and 1D building blocks in the form of clay platelets and nanofibrillar cellulose enhances the mechanical properties of a PVA matrix. Along similar lines, numerous studies on artificial nacre are based on embedding graphene oxide and/or ceramic structures into a soft and flexible polymer matrix^13^,^14^,^15^,^25^,^27^. In all these cases, tensile strength values in the range of 90 to 230 MPa as well as remarkable values in ultimate strain (6 to 55 %) are obtained, whereas the Young’s moduli (up to 6.9 GPa) are much lower than that of natural nacre (40 to 90 GPa)^6^,^7^. By contrast, the present V_2_O_5_ nanofibre-based paper comprising 0.5 wt% GO consists of a comparatively hard ceramic-based matrix in which softer GO is embedded. This “inverse” composition yields high tensile strength and good toughness combined with excellent stiffness. However, increasing the GO content to 2.5 or even further to 5.0 wt% GO increasingly reduces the mechanical performance. The decreasing values of tensile strength and Young’s modulus, in combination with the SEM cross-section images (Fig. 4c,d), are consistent with the observed out-of-plane softening (Fig. 3a). The influence of the GO nanosheets on the V_2_O_5_ nanofibre alignment during the self-assembly process is more pronounced at higher GO contents (5.0 wt%). It is concluded that locally disturbed alignment of V_2_O_5_ nanofibres and an increasing number of folded GO sheets (Supplementary Fig. S1) weaken the composite material and favor slipping of composite layers and pullout effects, leading to values of tensile strength (58 MPa) and Young’s modulus (16 GPa), which are even inferior to those of the V_2_O_5_ nanofibre- and GO papers. Comparing the composites’ values with other V_2_O_5_-based papers, such as a V_2_O_5_/diblock copolymer binary system (tensile strength of 24 to 26 MPa and Young’s moduli between 2.9 and 3.7 GPa)^45^, further demonstrates the superior mechanical performance of the present GO-containing, ternary system.

The difference between the mechanical properties associated with the in-plane and out-of-plane direction is a direct consequence of the highly anisotropic microstructure of the composites, i.e., the layered arrangement of the 1D V_2_O_5_ nanofibres and the 2D GO nanosheets, which are interconnected by hydrogen bonds and oxygen bridges. It is an intriguing observation that combining the stiff in-plane but compliant out-of-plane V_2_O_5_ nanofibres and GO nanosheets is able to enhance only the in-plane mechanical performance, but not the out-of-plane performance. For the in-plane case, such improvement over the V_2_O_5_ nanofibre- and GO papers is observed for a GO content of 0.5 and 2.5 wt%, as reflected by the strong increase of strength and moderate increase of the Young’s modulus. It can be attributed to a synergistic effect of the two nanostructured components, whose high in-plane stiffness ensures that also the pristine GO or V_2_O_5_ nanofibre papers display an excellent Young’s modulus of approximately 25 GPa. For the out-of-plane case, by contrast, the mechanical compliance of the components leads to a smaller Young’s modulus of the papers (9.9 GPa for V_2_O_5_ nanofibre paper and 3.7 GPa for GO paper, as determined by nanoindentation). In principle, incorporation of the soft and flexible GO sheets could have a beneficial effect, similar to many structural biomaterials such as nacre, where the addition of a small amount of a soft component strongly increases the mechanical performance^2^,^3^,^4^. However, this requires that the optimum thickness ratio of 10:1 between the soft and hard component^10^ is not exceeded. Hence, this result implies that that the addition of 0.5 wt% of GO already falls above this limit.

**Figure 5:**
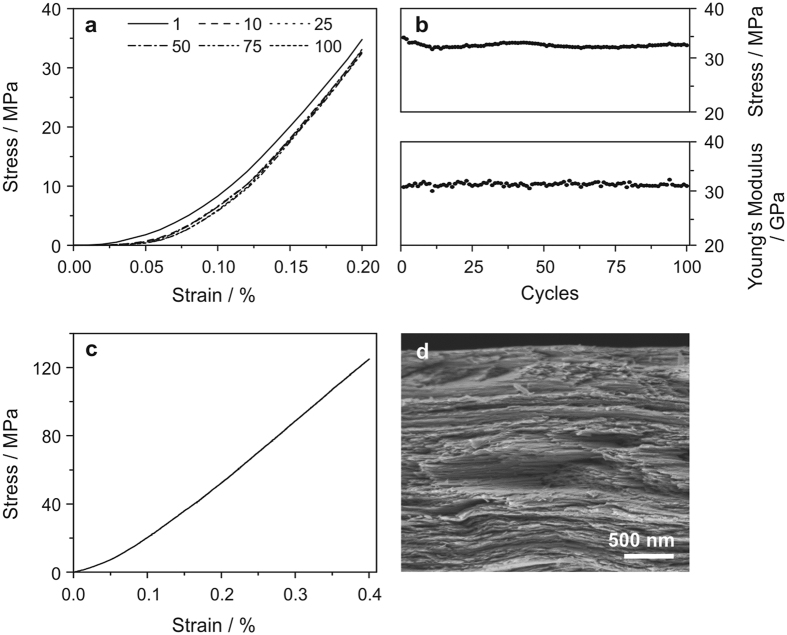
Mechanical cycling of a paper containing 0.5 wt% GO. (**a**) Nanotensile test loading curves of cycle 1, 10, 25, 50, 75 and 100. (**b**) Stress and Young’s modulus at a strain of 0.2% over 100 cycles. (**c**) Stress-strain curve and (**d**) SEM cross-sectional image of the paper after 100 cycles.

Based on the above results, repeated nanotensile tests were performed on the composite papers with 0.5 wt% GO, which showed the best mechanical performance, with the aim of investigating cycling stability of the composite material (Fig. 5). The maximum strain of 0.2 % was chosen based upon the ultimate strain at fracture, obtained from the destructive nanotensile tests (Fig. 4a). In order to evaluate the Young’s modulus, it was crucial to stretch the paper up to a point at which the stress-strain curve reaches a linear regime, but still below the measured ultimate strain (0.51 %). Repeated stretching of the paper up to a strain of 0.2 % led to a small, irreversible sample elongation after the first cycle, caused by a slight reorientation of the material along the applied mechanical load. The loading curves of the following cycles (number 10, 25, 50, 75 and 100) overlap (Fig. 5a). Stress and Young’s modulus show stable values of about 33 MPa and 32 GPa, respectively, over 100 measured cycles at 0.2 % strain (Fig. 5b), signifying a good cycling stability of the composite papers. In addition, nanotensile tests (until fracture) on samples, which were pre-stretched for 100 cycles, indicated that the cyclic loading does not damage the paper. Importantly, stress-strain curves and SEM cross-sectional images of composite papers with 0.5 wt% GO, before (Fig. 4a,b) and after 100 loading cycles (Fig. 5c,d), are almost identical.

**Figure 6:**
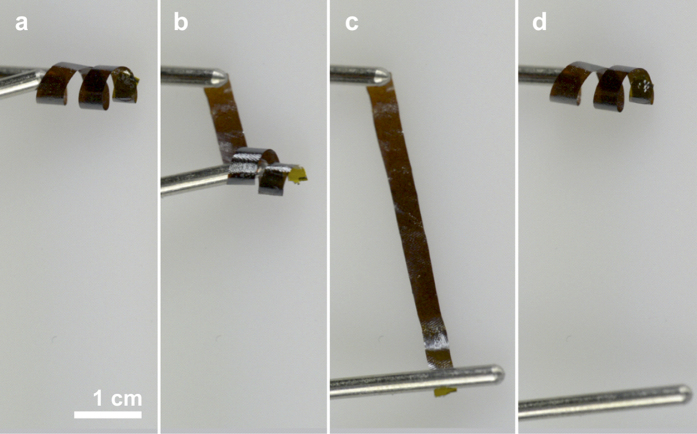
Macroscopic shape memory. (**a**–**c**) Unrolling of a coil made from a composite paper with 0.5 wt% GO. (**d**) After releasing the mechanical stress, the paper immediately re-assumes its macroscopic shape.

The remarkable mechanical performance and cycling stability of the composite material incorporating 0.5 wt% GO was visualized using macroscopic 3D structured papers (Fig. 6). A wet-shaped coil, produced from the originally flat paper, was stiff enough to maintain its new bulk structure, yet it was flexible enough to be easily deformed, for example by unrolling (Fig. 6a–c). After releasing the deformed papers, they immediately returned to their initial shape (Fig. 6d). Such mechanical deformation could be repeated multiple times without disturbing the micro- or macroscopic shape of the papers (Supplementary Video S1).

## Conclusion

In summary, we produced ternary V_2_O_5_ nanofibre-based composite papers *via* a low energy self-assembling approach. The combination of hydrated V_2_O_5_ nanofibres (1D) and GO nanosheets (2D) provides access to hierarchically layered microstructures with remarkable mechanical performance. This is enabled by the different Young’s moduli (9.9 GPa vs. 3.7 GPa, obtained by nanoindentation) of the components and their high aspect ratios. Maximum synergistic effects emerge for V_2_O_5_ nanofibre papers containing 0.5 wt% GO. Our findings show that the implementation of natural structure concepts into artificial, multifunctional materials is a valuable strategy to achieve a unique combination of mechanical properties. The free-standing papers’ high flexibility allows them to adapt and retain various shapes, like zigzag or coil. Moreover, they are stiff enough to memorize their micro- and macroscopic shapes even after repeated mechanical deformation. These properties are promising for applications such as actuators and mechanically stable, but flexible electrodes for advanced energy storage and conversion.

## Methods

### Preparation of the V_2_O_5_ nanofibre solution

In accordance with the sol-gel method pioneered by Livage^30^, the hydrated V_2_O_5_ nanofibres were synthesized by adding NH_4_VO_3_ (1 g) and Dowex 50WX8 50-100 ion-exchanger (10 g) to deionized water (200 ml). Subsequent storing the solution for 10 minutes in an oil bath at 80 °C, while stirring, induced the fibre formation, whereupon the solution turned dark red. After slow cooling, the solution was aged for 2 weeks under ambient conditions, leading to fibres with a length of up to 4 μm.

### Preparation of the GO nanosheet solution

GO nanosheets were synthesized *via* a modified Hummer’s method^32^. KNO_3_ (0.6 g) and graphite flakes (0.5 g, flake size 300 μm) were added to cooled 98% H_2_SO_4_ (23 ml), while stirring the acid. Slow addition of KMnO_4_ (3 g) initialized the oxidation of the graphite flakes. The mixture was then heated to 35 °C and held at that temperature for 6 hours. Dropwise addition of deionized water (40 ml) increased the temperature to 80 °C. This temperature was held for another 30 min. Finally, dilution of the mixture with deionized water (100 ml) and the addition of H_2_O_2_ (3 ml) afforded a bright yellow suspension, containing brownish flakes. Vacuum filtration and washing the oxidation product with deionized water, followed by centrifugation and mild sonication resulted in a clear, brown solution, containing monolayer GO sheets with a lateral dimension of several tens of micrometres.

### Preparation of the composite papers

The aqueous V_2_O_5_-GO solutions were produced by sonication-assisted mixing of V_2_O_5_ nanofibre solution and GO nanosheet solution at a volume ratio of 10 to 1, with varying the GO concentration. Self-assembly of the mixed solutions onto silicon wafers under ambient conditions led to flat thin-films with a GO content of 0.5, 2.5 and 5.0 wt% and a thickness of about 2.5 μm, analogous to the procedure already described for the initial V_2_O_5_ nanofibre papers^29^. The dry film was then lifted-off by immersing the substrate in a deionized water bath, followed by drying on a sieve.

### Structural characterization

Structural analysis of the papers was carried out with a Zeiss Merlin SEM operated at 1.5 kV and a Zeiss Libra 200 TEM using 80 kV acceleration voltage. Further investigations were done by D8 Discover XRD (Bruker AXS), using a Cu Kα radiation in parallel beam geometry. Surface sensitive techniques comprised tapping mode AFM (Bruker Multimode 8 with Nanoscope V control unit) and Raman spectroscopy (Horiba LabRam) equipped with a red laser (633 nm).

### Mechanical characterization

Out-of-plane mechanical characterization was performed by depth-resolved nanoindentation of thin films that were still attached to the silicon wafers. Indents were created with the Berkovich tip of a Nanoindenter XP (Keysight Technologies) in continuous stiffness mode to a maximum depth of 500 nm. In-plane mechanical properties were investigated with a Nano Bionix (Keysight Technologies), which has a load resolution of 50 nN. Prior to measurements, the samples were prepared by cutting strips of the free-standing papers and gluing them on a standardized cardboard frame, which was inserted into the setup. All measurements were carried out on samples with a gage length of about 10 mm and a strain rate of 1·10^−4^ mm s^−1^.

### Shaping the composite papers

Cut strips of the papers were placed on the surface of a deionized water bath. A straight mesh material was submerged underneath the swimming paper in order to fish it out carefully, so that it adapts the mesh’s shape. Subsequently coiling up the mesh and allowing the paper to dry in that state yielded the retained coil structure.

## Additional Information

**How to cite this article**: Knöller, A. *et al*. Strengthening of Ceramic-based Artificial Nacre via Synergistic Interactions of 1D Vanadium Pentoxide and 2D Graphene Oxide Building Blocks. *Sci. Rep.* **7**, 40999; doi: 10.1038/srep40999 (2017).

**Publisher's note:** Springer Nature remains neutral with regard to jurisdictional claims in published maps and institutional affiliations.

## Supplementary Material

Supplementary Information

Supplementary Video S1
