# Supplementary material for: Strengthening of Ceramic-based Artificial Nacre via Synergistic Interactions of 1D Vanadium Pentoxide and 2D Graphene Oxide Building Blocks
Source: Sci Rep. 2017 Jan 19;7:40999. doi: 10.1038/srep40999 (PMC5244477; doi:10.1038/srep40999)
Supplement: Supplementary Information [file srep40999-s1.pdf]

Supplementary Information

Strengthening of Ceramic-based Artificial Nacre via Synergistic Interactions of 1D Vanadium Pentoxide and 2D Graphene Oxide Building Blocks

Andrea Knöller,[1] Christian P. Lampa,[1] Felix von Cube,[2] Tingying Helen Zeng,[3,4] David C. Bell,[2] Mildred S. Dresselhaus,[3,5] Zaklina Burghard[1,] and Joachim Bill[1]*

[1]Institute of Materials Science, University of Stuttgart, Heisenbergstr.3, 70569 Stuttgart, Germany

E-Mail: zaklina.burghard@imw.uni-stuttgart.de

[2]John A. Paulson School of Engineering and Applied Sciences, Harvard University, Cambridge, MA 02138, USA

[3]Department of Electrical Engineering and Computer Sciences, Massachusetts Institute of Technology, Cambridge, MA 02139, USA

[4]Department of Chemistry and Chemical Engineering, School of Chemical Engineering and Environment, Beijing University of Technology, Beijing, 100124, P.R. China

[5]Department of Physics, Massachusetts Institute of Technology, Cambridge, MA 02139, USA

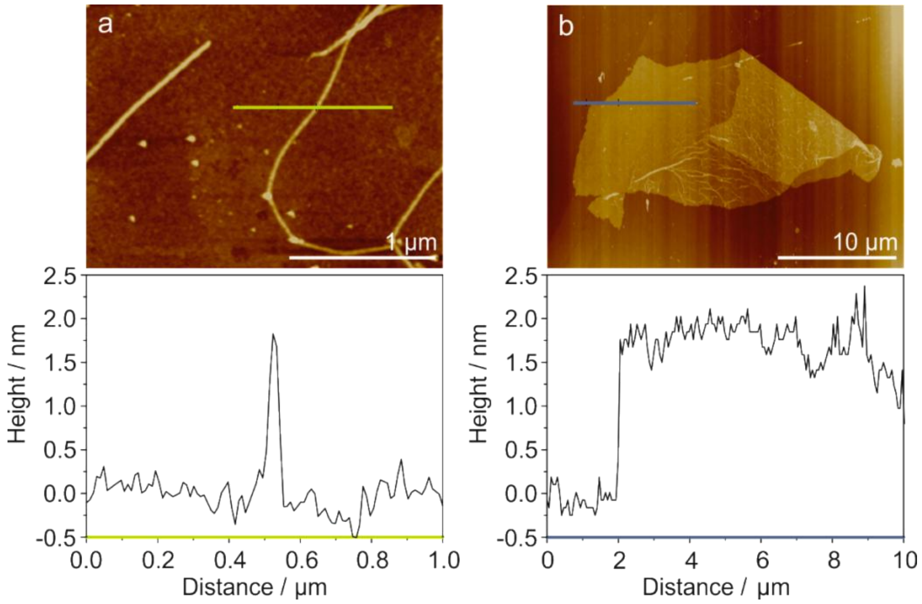

Figure S1|AFM height images and profiles. a, a hydrated V_2O_5 nanofibre and **b,** GO nanosheets. Both materials exhibit a height of about 1.5 nm.

Sample preparation and compositional details

All samples were fabricated using the same volume ratio between V_2O_5 nanofibre solution and GO nanosheet solution. While the concentration of the V_2O_5 nanofibre solution was kept constant, the GO nanosheet solution was adjusted by diluting the stock solution to obtain the desired final GO content. Concerning the water content, the initial V_2O_5 nanofibre papers appeared to have a water content of about 12.5 wt%, which corresponds to 1.4 H_2O molecules per V_2O_5.[1] This content is correlated to the material's interlayer distance of 1.04 nm, which was extracted from XRD peak position analysis. The fabrication conditions as well as the resulting thickness and interlayer distances (between 1.03 and 1.05 nm) of the GO-containing composite papers are nearly the same as for the V_2O_5 nanofibre papers. It can be concluded that the water content of the V_2O_5 nanofibres in all papers are also alike.

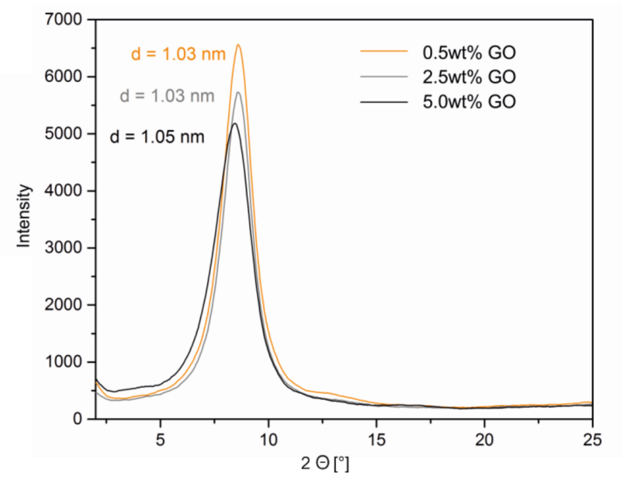

Figure S2|XRD peak position analysis of composite papers. All papers exhibit an interlayer distance between 1.03 and 1.05 nm.

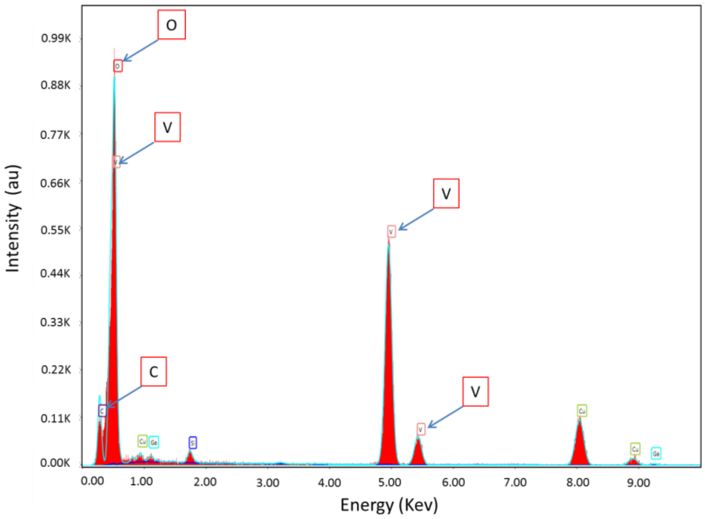

Figure S3|Energy-dispersive X-ray spectroscopy (EDS) data recorded by cross-sectional TEM of the composite lamella. The vanadium-, oxygen- and carbon-peaks are assigned to the present components (hydrated V_2O_5 and GO). In addition, there are peaks due to the silicon from the substrate, the copper of the TEM grid, and gallium from the FIB beam used to cut the lamella.

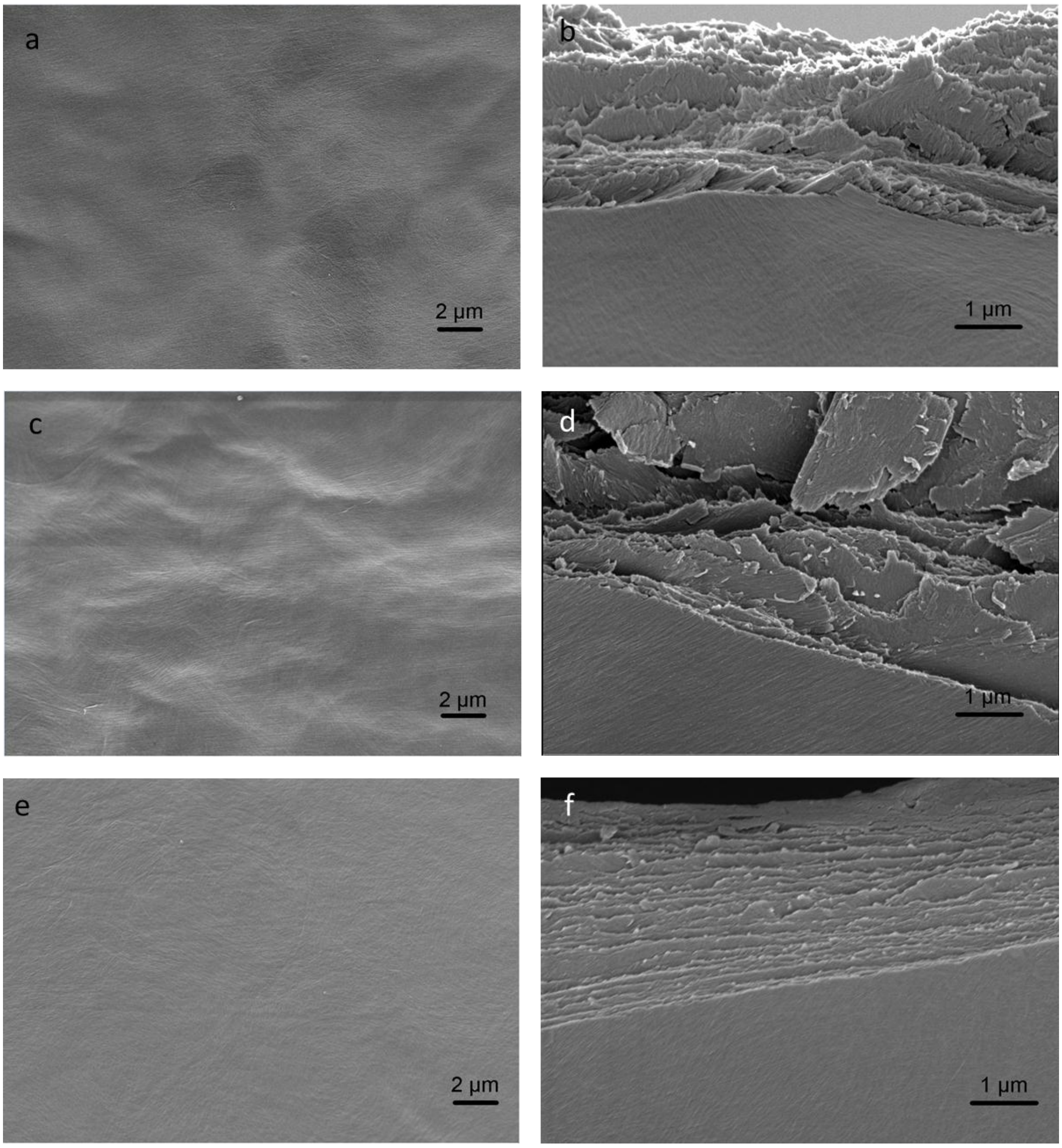

Figure S4| SEM images illustrating the alignment of the V_2O_5 fibres on the surface of the composites. a, 0.5 wt%; **c,** 2.5 wt% and **e,** 5.0 wt% GO, respectively, and the fracture morphology of the composites with **b,** 0.5 wt%; **d,** 2.5 wt% and **f,** 5.0 wt% GO, respectively.

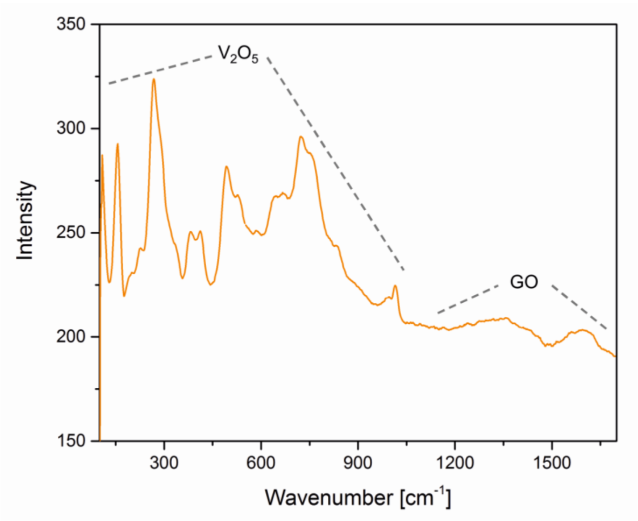

Figure S5|Raman spectra of a paper containing 0.5 wt% GO paper. Bands in the lower wavenumber region are assigned to the hydrated V_2O_5 matrix material, whereas the two bands at 1348 and 1592 cm^{-1} are assigned to the D and G band of GO, respectively.

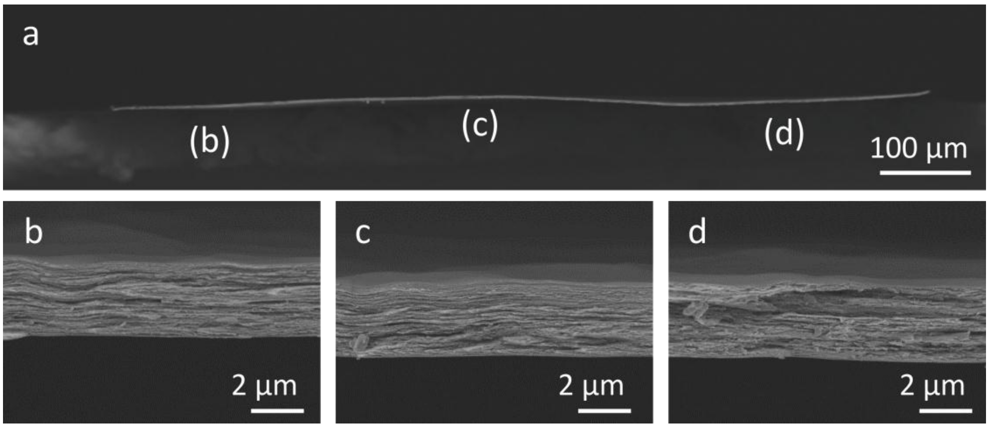

Figure S6|SEM image of a paper's complete cross-section. a, overview of different positions **b-d,** showing the uniformity of the paper's thickness.

Table S1: **Mechanical properties of the composite materials, as determined from tensile tests.**

V_2O_5 papers with	Tensile Strength [MPa]	Young's Modulus [GPa]	Ultimate Strain [%]	Toughness [kJ/m³]
**0.5 wt% GO**	139 ± 8	33 ± 6	0.51 ± 0.11	325 ± 69
**2.5 wt% GO**	87 ± 16	27 ± 4	0.41 ± 0.06	156 ± 48
**5.0 wt% GO**	58 ± 9	16 ± 4	0.47 ± 0.12	125 ± 54

Video S1|Visualization of the macroscopic shape memory. The combination of stiffness and flexibility allows to repeatedly unroll a coil made from a composite paper with 0.5 wt% GO. After mechanical deformation the paper immediately returns into its coil-like shape.

References

1. Burghard, Z. *et al.* Hydrogen-Bond Reinforced Vanadia Nanofiber Paper of High Stiffness. *Adv. Mater.* **25,** 2468–2473 (2013).
